# Supplementary material for: Hazard Function Analysis of Recurrence in Patients with Curatively Resected Lung Cancer: Results from the Japanese Lung Cancer Registry in 2010
Source: Cancers (Basel). 2022 Oct 19;14(20):5119. doi: 10.3390/cancers14205119 (PMC9600058; doi:10.3390/cancers14205119)
Supplement: Supplementary file 1 [file cancers-14-05119-s001.zip › cancers-1953614-supplementary.pdf]

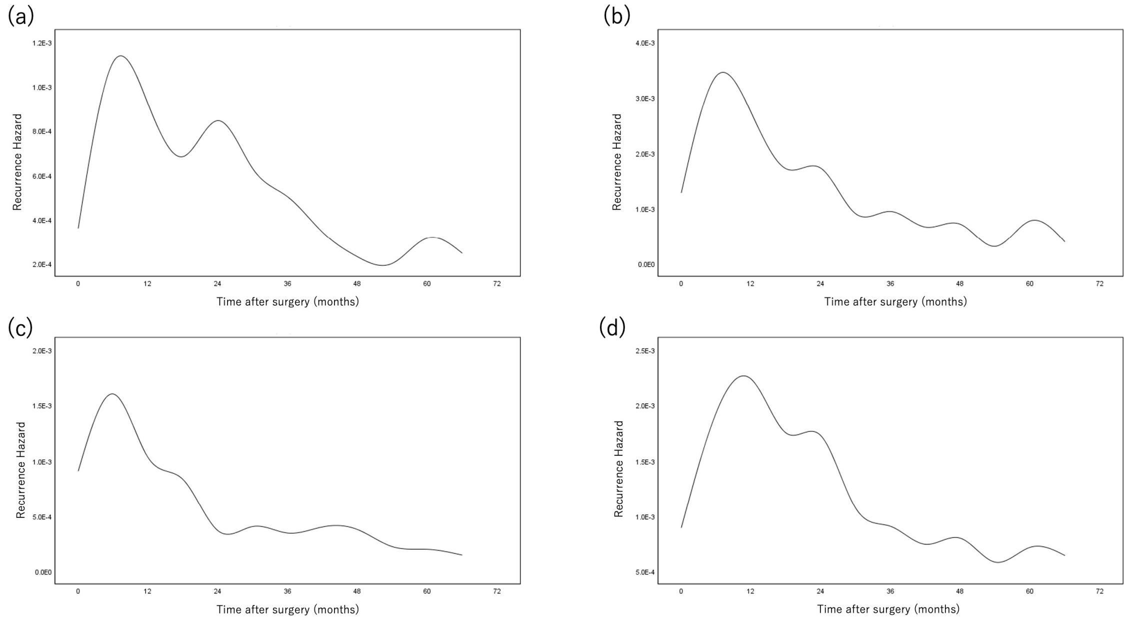

Figure S1. Recurrence hazard functions for recurrence at the surgical stump (**a**) mediastinal or hilar lymph nodes (**b**), pleural dissemination (**c**), and lung (**d**).

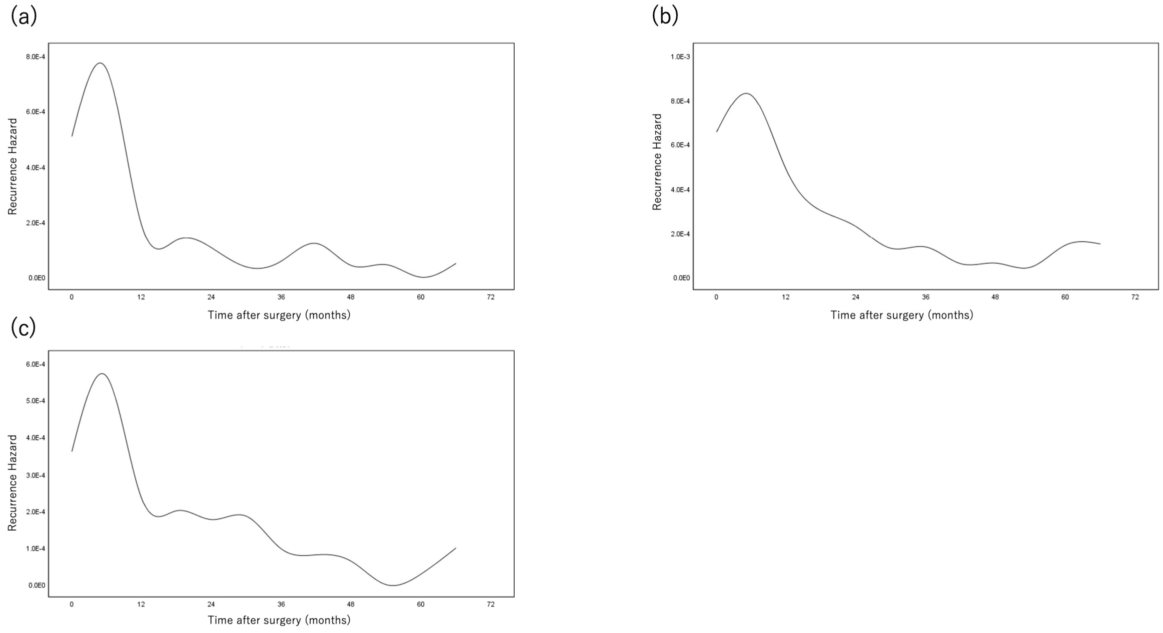

Figure S2. The recurrence hazard function for recurrence at the adrenal grand (**a**), liver (**b**), and extrathoracic lymph nodes (**c**).

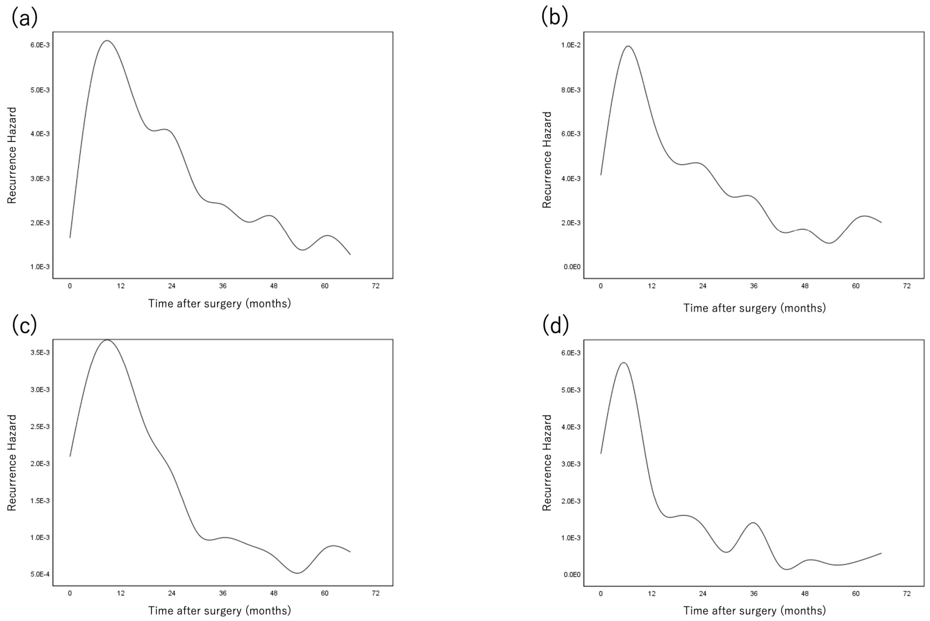

Figure S3. The recurrence hazard functions for intrathoracic recurrence in patients with (**a**) adenocarcinoma and (**b**) squamous cell carcinoma; and the recurrence hazard functions for extrathoracic recurrence in patients with (**c**) adenocarcinoma and (**d**) squamous cell carcinoma
